# Ultrasound delivery of Surface Enhanced InfraRed Absorption active gold-nanoprobes into fibroblast cells: a biological study via Synchrotron-based InfraRed microanalysis at single cell level

**DOI:** 10.1038/s41598-019-48292-0

**Published:** 2019-08-14

**Authors:** F. Domenici, A. Capocefalo, F. Brasili, A. Bedini, C. Giliberti, R. Palomba, I. Silvestri, S. Scarpa, S. Morrone, G. Paradossi, M. D. Frogley, G. Cinque

**Affiliations:** 1 Dipartimento di Scienze e Tecnologie Chimiche, Università degli Studi di Roma “Tor Vergata”, Rome, Italy; 2 Dipartimento di Fisica, Università degli Studi di Roma “Sapienza”, Rome, Italy; 3 Dipartimento Innovazioni Tecnologiche e Sicurezza degli Impianti, Prodotti e Insediamenti Antropici (DIT), INAIL, Monteporzio Catone, Rome, Italy; 4 Dipartimento di Medicina Molecolare, Università degli Studi di Roma “Sapienza”, Rome, Italy; 5 Dipartimento di Medicina Sperimentale, Università degli Studi di Roma “Sapienza”, Rome, Italy; 6 MIRIAM beamline B22, Diamond Light Source, Harwell Campus, Chilton-Didcot, OX11 0DE UK

**Keywords:** Nanoscale biophysics, Diagnosis

## Abstract

Ultrasound (US) induced transient membrane permeabilisation has emerged as a hugely promising tool for the delivery of exogenous vectors through the cytoplasmic membrane, paving the way to the design of novel anticancer strategies by targeting functional nanomaterials to specific biological sites. An essential step towards this end is the detailed recognition of suitably marked nanoparticles in sonoporated cells and the investigation of the potential related biological effects. By taking advantage of Synchrotron Radiation Fourier Transform Infrared micro-spectroscopy (SR-microFTIR) in providing highly sensitive analysis at the single cell level, we studied the internalisation of a nanoprobe within fibroblasts (NIH-3T3) promoted by low-intensity US. To this aim we employed 20 nm gold nanoparticles conjugated with the IR marker 4-aminothiophenol. The significant Surface Enhanced Infrared Absorption provided by the nanoprobes, with an absorbance increase up to two orders of magnitude, allowed us to efficiently recognise their inclusion within cells. Notably, the selective and stable SR-microFTIR detection from single cells that have internalised the nanoprobe exhibited clear changes in both shape and intensity of the spectral profile, highlighting the occurrence of biological effects. Flow cytometry, immunofluorescence and murine cytokinesis-block micronucleus assays confirmed the presence of slight but significant cytotoxic and genotoxic events associated with the US-nanoprobe combined treatments. Our results can provide novel hints towards US and nanomedicine combined strategies for cell spectral imaging as well as drug delivery-based therapies.

## Introduction

An ever-growing interest has been focused on the ability of ultrasound (US) of transmitting mechanical stimuli to cells in order to trigger a temporary alteration of the plasma membrane permeability^1,2^. This phenomenon, known as reparable sonoporation (SP), can be exploited to non-invasively target mechanical energy into biological organisms in favour of novel drug and gene delivery strategies^3,4^. For this reason, the effects of SP are now intensively studied in nanomedicine offering a pivotal chance to design improved anticancer strategies for the targeting of nanocarriers through the cytoplasmic membrane^5^. In the latter framework, recent advances in nanoscience and nanotechnology have paved the way for engineering several different diagnostic and therapeutic delivery vectors showing key roles in selectively seeking out, tracing and promoting cell by cell ablation of tumour tissues^6–16^.

Nevertheless, the progress to this end suffers of lacking knowledge on the potential adverse effects of both US and nanomaterials interacting with biological systems. It is important to emphasise in this respect that membrane SP may affect cell osmosis, activate inflammatory pathways and even weaken the endothelium and skin innate immunity protection with the risk that submicrometric agents enter the organism. Generally, concomitant bioeffects have been reported in literature^17–19^ on cells underwent US, depending on the exposure parameters (i.e., frequency, intensity and time). The lack of cellular recovery from such alterations leads to possible insurgence of cytotoxic and genotoxic events^20,21^. Herein we focused on the well-established murine fibroblasts cell line, NIH-3T3, being fibroblast cells predominant in connective tissue. This cell line has been successfully employed in studying US-induced bioeffects, and this can help us to compare and to interpret our results reliably^17,18,22^.

We recently pointed out the experimental conditions to obtain reversible plasma membrane permeability enhancement on NIH-3T3 fibroblast and HaCaT keratinocytes cells at very low intensity megasonic fields (in the subcavitation regime)^17,18,23^. According to the BiLayer Sonophore model^2^, these effects were related to transient alterations of the plasma membrane^17,23^ allowing efficient internalisation of molecules with Stokes diameters of the order of magnitude of 10 nm, observing no severe cell damage^18^. Taking advantage of this latter finding, the internalisation of nanomaterials *in vitro* by SP has been considered herein.

Recently, hybrid gold nanoparticles, nano-hydrogels, and mesoporous platforms have been employed as invaluable nano-soldiers in targeting cancer, showing good specific area and versatility in carrying drugs and exerting inhibitory effects on tumour cells. Specifically, stimuli-responsive (e.g., via pH, thermo-optical inputs) carriers such as chitosan oligosaccharide grafted halloysite nanotubes^11^, poly(lactic-co-glycolic acid)-based drug reservoir platforms^17,24^, polydopamine-modified mesoporous silica nanocarriers^19,23^, black phosphorus nanosheets, poly(ethylene glycol)- and borate-coordination polymer-coated polydopamine nanoparticles^21^, have exhibited promising loading efficiency of chemotherapeutics (e.g. doxorubicin, docetaxel), dose-limiting side effects, reduced toxicity/efficacy ratio, and selectivity towards tumour tissue (e.g. breast, cervical cancer), even in synergistic chemotherapy, photothermal and gene combined approaches^25^.

Among all the different nanomaterials that can be used as both carriers and probes, gold nanocolloids (AuNPs) have received much biomedical attention because of their high surface-to-volume ratio, easy biofunctionalisation, chemical stability, and unique ability of providing local amplification of electromagnetic fields by resonant collective electronic oscillations (named localised surface plasmons)^25,26^. Specifically, the plasmonic-mediated capability of AuNPs to enhance the infrared absorption cross-section of specific organic and biological molecules located in proximity of their surface^24,27–30^ is shedding new light on the development of novel ultrasensitive detection and specific signalling methodologies^31–33^. The phenomenon, known as Surface Enhanced Infrared Absorption (SEIRA), consists in the enhancement of the optical field confined at the surface of the plasmonic particle when illuminated by resonant infrared light^34^. The resonant absorption due to localised surface plasmons can be tuned by a series of AuNPs parameters such size in the nanometre scale, shape, self-assembling, and dielectrics of surrounding environment^35–37^. Furthermore, there is also a chemical effect which contributes to the SEIRA enhancement, related to transition dipole moment variations of the molecules adsorbed onto a nanostructured surface^24,27,28,30^. SEIRA spectroscopy presents some unique features, with respect to the better-known Surface Enhanced Raman Scattering (SERS)^28,38,39^ and fluorescence spectroscopy, as a sensitive molecular detection tool in biological matter. In this respect, the infrared absorbance cross-section values of molecules are usually significantly higher than those exhibited by Raman scattering, yielding an overall SEIRA sensitivity comparable to that of SERS. Moreover, infrared detection is not as destructive as fluorescence spectroscopy and resonant Raman.

In this framework, several reports have shown that AuNPs of suitable dimensions can be easily functionalised with the hetero-bifunctional linker 4-aminothiophenol (4ATP), to produce an efficient IR marker 4ATP-AuNP conjugate, characterised by several intense SEIRA vibration modes ranging from 1700 to 900 cm^−1^ ^24,27,29^. 4ATP presents the advantage of exposing a free amino group (-NH_2_) outside the core-shell system, which can be employed for further conjugations with different molecules of biological interest^23,40^. Despite this, the literature describing cell probing by SEIRA, and in particular on 4ATP-AuNPs, is lacking or missing until now. Moreover, any biologically harmful side- or after-effects of this promising class of nanoprobes remain rather obscure to date^41^.

Synchrotron Radiation Fourier Transform Infrared micro-spectroscopy (SR-microFTIR) has emerged as a valuable analytical tool for the monitoring of biochemical changes induced by various external agents at the single cell level^42^. The signal-to-noise ratio, with the same set up and similar measurement conditions as this work, on single cell by microFTIR, is between 9 to 30 times larger by SR vs. conventional source, depending on the slit size selected at the microscope^43^. In this work, we relied on the high sensitivity that SR-microFTIR offers in single cell signalling to recognise the spectroscopic and biochemical influence of SEIRA-activated AuNPs which entered the cell by SP^44^. Experimental efforts have been successfully conducted to study the US-mediated internalisation of 4ATP-AuNPs within single NIH-3T3 cells, thus shedding new light on the advantageous synergistic use of SR-microFTIR spectroscopy and SEIRA. The possible biological and toxicological implications resulting from the nanoprobe internalisation have been investigated by coupling the spectroscopic analyses with viability and chromosomal damage end point assays by means of fluorescence microscopy and flow cytofluorimetry.

Taking care of the risk-to-benefit balancing of US-based treatments our results may provide new insights for designing novel optical screening of theranostic relevance.

## Materials and Methods

### Cell culture

NIH-3T3 murine fibroblasts were cultured as a monolayer in a humidified atmosphere with 95% air and 5% CO_2_ at 37 °C in Dulbecco’s modified Eagle’s medium (Sigma–Aldrich) with 10% fetal bovine serum (Sigma–Aldrich), 1% penicillin/streptomycin (Sigma–Aldrich) and 1mM L-glutamine (Sigma–Aldrich). Cells were seeded in 35 mm tissue culture dishes (Falcon^®^ Easy Grip^TM^) containing 3 ml of medium and grown to 90% confluence (7~9 × 10^5^ cells per plate) in a tissue culture incubator at 37 °C, 5% CO_2_ for about 48 hours. For apoptosis tests the cells were treated with the standard apoptosis inducer staurosporine (Sigma) that was dissolved in DMSO (Sigma) in stock solution of 1 mM and used at the concentration of 5 µM diluted in culture medium for 6 hours.

### Nanoprobe assembly and characterisation

The 4ATP-AuNPs nanoprobe considered here consists of a *core-shell* system made up of AuNPs (Sigma-Aldrich), with size ranging between 2 and 20 nm, functionalised with the aromatic molecule 4ATP (Sigma-Aldrich). The AuNPs are dispersed at the concentration of 1.2 nM (number density 7.20 × 10^11^ ml^−1^) in a 0.1 mM PBS solution. The morphology and size of AuNPs were analysed by field emission scanning electron microscopy (FE-SEM), absorption spectroscopy, Dynamic Light Scattering (DLS) and ζ-potential measurements. Representative data for 20 nm AuNPs are shown in Section S2 of the ESI.

Following Domenici *et al*., the AuNPs were added 1:1 to a 4ATP solution in ethanol^45^. The 4ATP final concentration (0.01 g/l) was in large excess with respect to the minimum needed to achieve a full coverage of the AuNP surfaces (e.g. 3900 molecules of 4ATP for AuNPs with 20 nm size). The solution was incubated for 3 hours at room temperature (RT) and the excess of 4ATP was removed by 24 hours dialysis against Milli-Q water under continuous gentle stirring using a filter with a cutoff of 1 KDa.

The nanoprobe assembly was monitored by UV-Visible absorption spectroscopy, DLS and ζ-potential. UV-Visible measurements were performed using a Jasco v-570 double ray spectrophotometer, with a resolution of 0.1 nm. For DLS measurements, a Malvern NanoZetaSizer apparatus, equipped with a 5 mW He-Ne laser (Malvern Instruments Ltd), was employed. Experiments were performed at T = 25 °C. The autocorrelation functions of the scattered light intensity were collected at an angle of 173° and analysed with the CONTIN algorithm, to obtain the size distributions. The reported values are the average of several measurements and are obtained from intensity weighted distributions. ζ-potential was obtained by the Phase Analysis Light Scattering (PALS) technique using the same Malvern NanoZetaSizer apparatus. The measurements were performed employing a palladium electrode dip cell ZEN 1002 (Malvern, UK) and the ζ-potential values were derived from the sample electrophoretic mobility using the Smoluchowski relation.

### Atomic Force Microscopy and FE–SEM

Nanoprobes deposited onto silicon substrates were characterised by Atomic Force Microscopy (AFM) and Field Emission Scanning Electron Microscope (FE-SEM) at Sapienza Nanoscience & Nanotechnology Laboratories (SNN-Lab) of the Research Center on Nanotechnology Applied to Engineering of Sapienza University (CNIS). Samples were prepared by drop casting and dried at RT.

For AFM, a Dimension Icon Bruker microscope was employed in tapping mode, with a scan rate of 0.5 Hz, using a cantilever with a spring constant of 42 N/m and a tip with a nominal radius of curvature of 8 nm. Data analysis was performed by Gwyddion software, version 2.50. SEM images were recorded using a Zeiss Auriga 405 Microscope.

### US exposure setup and cell treatment protocol

For studies of mechanical poration of the cell plasma membrane by US exposure, a medical device (Nuova Elettronica, Rome, IT) consisting of a submersible piezoceramic circular unfocused transducer (6 cm diameter) tuned at 1 MHz was used. The employed exposure setup was described in detail by Domenici *et al*.^17^. Briefly, the ultrasonic transducer was placed at the bottom of a tank 30 × 30 × 30 cm filled with degassed water at a fixed temperature of 25 °C. A special cell for micro-volumes (400 μl filling volume), hermetically lidded, was positioned in contact with the water surface coaxially aligned with the US transducer. The calibration of the US field was performed measuring the acoustic intensity by varying the source-sample distance (SSD), i.e. the distance between the transducer and the lower surface of the plate, by means of a needle hydrophone (S.N. 1470, Precision Acoustics) of 1 mm diameter with sensitivity at 1 MHz of 1670.4 mV/MPa (±14%). We selected a SSD of 5 cm, at which the resulting US field was stable and reproducible. The intensity of the acoustic field was analysed in terms of ‘spatial peak temporal average intensity’ (I_spta_), which represents the spatial maximum of the temporal averaged intensity measured for the whole period of treatment.

NIH-3T3 cells grown in Petri plates were detached by Trypsin-EDTA solution (EuroClone S.p.A.) and centrifuged (1200~1500 rpm). The pellet was re-suspended in 200 µl of PBS and added to 200 µl of 4ATP-AuNPs dispersion or Milli-Q water. The sample was then transferred in a cell for micro-volumes and treated by US in a continuous sinusoidal regime with I_spta_ = 16 mW/cm^2^ (significantly below the threshold of the cavitation regime of 100 mW/cm^2^) and exposure times of 15 and 30 minutes. According to Domenici *et al*.^18^, such exposure conditions allow internalisation of particles with size of tens of nanometers. After SP treatment a restoring time of 10 minutes was scheduled for the resealing of the pores in the cell membrane^18^.

For *in vitro* biological assays, US and 4ATP-AuNPs treatments were performed at the same doses employed for the microFTIR investigation, maintaining the cell culture in adhesion to avoid affecting the cell metabolism.

### FTIR measurements

Preliminary transmission FTIR measurements were performed to characterise the IR signal of the nanoprobes prepared using AuNPs with different sizes. Samples were dried onto CaF_2_ substrates and spectra were acquired using a FTIR/410 Jasco spectrometer equipped with a conductive ceramic coil mounted in a water-cooled copper jacket source, a KBr beamsplitter and a TGS detector with a spectral resolution of 4 cm^−1^. The optical path was purged continuously with gaseous nitrogen.

SR-microFTIR measurements were performed at Diamond Light Source Synchrotron (Oxfordshire, UK) at the MIRIAM beamline B22^46^. A Bruker Vertex 80 V in vacuum FTIR with resolution 4 cm^−1^ was utilised. Each spectrum was acquired accumulating 256 scans. A Hyperion 3000 microscope was used for microFTIR in the 700~4000 cm^−1^ spectral range by means of a 36 × Cassegrain objective and condenser optics for transmission mode. 2D maps were acquired by a 15 µm × 15 µm microbeam and step of 5 µm (i.e. oversampling 3 times for optimal results), scanning areas of 60 µm × 60 µm up to 80 µm × 80 µm. Cell spectra acquisition was optimised further by repeating 3 measurements with a shift of 5 µm in order to have a better representation of the whole cell IR spectrum, and avoiding e.g. focusing on the cell nucleus or periphery signals only.

Measurements were performed on the following samples deposited on silicon substrates: i) nanoprobes made up of 20 nm AuNPs; ii) untreated cells; iii) cells exposed for 15 and 30 minutes to 20 nm 4ATP-AuNPs at 25 °C; iv) cells sonicated (15 and 30 minutes); v) cells sonicated in the presence of 20 nm 4ATP-AuNPs (exposure condition of point iv). For samples ii-v, spectra were collected on at least 20 different cells to obtain significant statistics. The cell samples were prepared according to the following procedure: the cells were dispersed in 10 ml of PBS and centrifuged, thereafter 50 µl of pellet were deposited on sterile hydrophilic silicon slides and desiccated at 37 °C. To remove excess buffer, a drop of solvent (90% Milli-Q water and 10% PBS to prevent rupture of the cell membranes) was deposited on the desiccated cells and then quickly removed by gaseous nitrogen flow. Afterwards samples were further desiccated for 3 hours.

All the spectra are presented after a baseline subtraction and a smoothing procedure by the Savitzky-Golay algorithm (9 points) performed using Origin 8.1 software. For sake of clarity some of them are normalised to the maximum absorbance as indicated in the corresponding caption. The frequencies of the main IR and SEIRA peaks were estimated by a Gaussian band fitting procedure.

### Cytotoxic test: Annexin V/propidium iodide apoptosis assay

AnnexinV/Propidium Iodide (PI) assay was used to analyse the cell death according to the following protocol. After the treatment, cells were dispersed in Dulbecco’s PBS and transferred in Falcon tubes for centrifugation. The pellet was then suspended in 1 ml of buffer solution at pH = 7.4 (named binding buffer: 10 mM of Hepes - NaOH, 140 mM of NaCl, 2.5 mM of CaCl_2_) filtered by sterile pore filter with cut-off of 0.2 μm, taking care of maintaining a cell number density of about 2~5 × 10^5^ cells/ml to ensure their detection by flow cytometry. A cell suspension volume of 195 μl was mixed with 5 μl of FITC-labeled AnnexinV in buffer solution (pH 7.4) made of 50 mM of TRIS, 100 mM of NaCl, 1% of BSA, 0.02% of Sodium Azide, followed by 10-minutes incubation at RT in the dark. Immediately after incubation, the solution was dispersed in Hepes (final volume of 2 ml) and centrifuged. The cell pellets were re-dispersed in 400 μl of the binding buffer and just before flow cytometry reading, 10 μl of PI stock solution (100 μg/ml in PBS) was added^18^. The experiments have been conducted in triplicate.

Flow cytometry analysis was performed by flow cytometer FACSCalibur (BD Biosciences, Singapore) endowed of Argon ion and visible red diode lasers at 488 nm and 635 nm wavelengths, respectively. Fluorescence emission was detected at 530 ± 15 nm (AnnexinV) and 585 ± 21 nm (PI), respectively; for each sample 10000 events were acquired. The results were analysed by CellQuest software (Becton Dickinson) and reported as percentage of positive cells (% gated).

### Cytotoxic test: Cleaved Caspase-3 immunofluorescence

5000 cells in 500 µl of medium were plated in 8-well cell chamber slides (Nunc, Naperville, IL, U.S.A.). After treatment cells were rinsed with PBS-Ca/Mg and fixed at 4 °C by 20 minutes incubation with 4% buffered paraformaldehyde (Sigma). The cells were therefore incubated for 1 hour at RT, first with 3% (w/v) solution of BSA, and then with a specific rabbit polyclonal primary antibody against cleaved caspase-3 (Cell Signaling Technology) diluted 1:100. Thereafter, the cells were washed twice with PBS and further treated for 1 hour with the secondary anti-rabbit antibody labelled with Alexa Fluor 594 diluted 1:400. The cells were then doubly rinsed with PBS, and nuclear staining was successfully achieved after 5-minutes treatment of 10 µg/ml of Hoechst 33342 (Sigma-Aldrich) in PBS solution. For microfluorescence investigation the cells were mounted with ProLong anti-fade reagent (Molecular Probes) and analysed by an Olympus BX52 fluorescence microscope, equipped with combination of excitation filter (540 nm) and emission filter (580 nm) for Alexa Fluor 594 (red), and with UV 330–380 nm and 420 nm filter sets for Hoechst (blue). The images were acquired and analysed by IAS 2000 software.

### Genotoxic test: Cytokinesis-block micronucleus assay

The genomic instability at chromosomal level was evaluated by Cytokinesis Block MicroNucleus (CBMN) assay. The cells (8 replicas per treatment) were added with 6 μg/ml Cytochalasin B (Sigma-Aldrich) immediately after treatment and grown for 24 hours to accumulate bi-nucleated cells. Afterwards the cells were centrifuged and suspended in a preheated hypotonic solution of 75 mM KCl and fixed using Carnoy’s solution (acetic acid:chloroform:methanol,1:3:6 v/v/v). The fixed cells were dropped onto pre-cleaned microscope slides and stained by adding 10 mg/ml of 4’,6’-diamidino-2-phenylindole (DAPI, Sigma, St. Louis, MO, USA) in antifade solution (Vector Laboratories, Burlingame, CA, USA)^19^. Micronuclei were scored at 1000x total magnification by means of Fluorescence Microscope Zeiss, with ApoTome (Axio Imager Z1 Stand).

### Statistical analysis

All experiments were performed at least in triplicate and the obtained results were provided as average value ± standard deviation. All results were assessed via one-way variance analysis (ANOVA) using SPSS 16.0 software, together with Tukey’s honest significance test. The level of significance was established at: *p < 0.05, **p < 0.01, ***p < 0.001 and ****p < 0.0001 as extreme statistical significance^18,19^.

## Results and Discussion

In this section we report the results of the analysis at the single cell level concerning the interaction between NIH-3T3 and our plasmonic nanoprobe. The uptake of 4ATP-AuNPs was triggered by applying 1 MHz US fields in the subcavitation regime, with I_spta_ = 16 mW/cm^2^ for 15 or 30 minutes. Using specific probes of membrane integrity, it has been recently demonstrated that within these conditions the plasma membrane permeability of NIH-3T3 cells can be temporarily altered, thus allowing the efficient internalisation of nanovectors with proper dimensions^17,18,23^.

**Figure 1:**
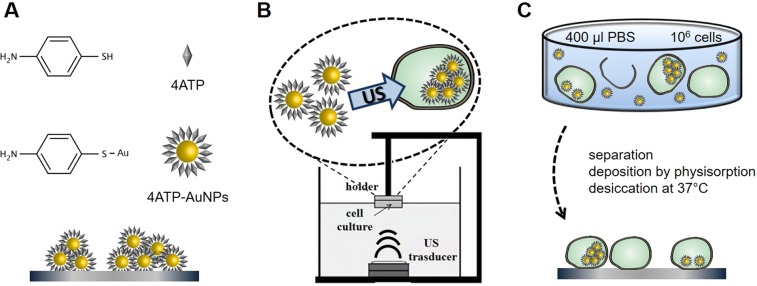
Scheme of the protocols employed for the SR-microFTIR analyses. (**A**) the nanoprobe assembling was obtained by functionalising the AuNPs with the IR marker 4ATP; spectra were acquired after depositing the nanoprobes onto silicon wafers. (**B**) the internalisation of the nanoprobes within NIH-3T3 cells was triggered by low-intensity 1 MHz US; the exposure setup employed consists of a water filled tank, a transducer positioned at its bottom and a micro-volume cell holder vertically aligned with the transducer; the sonication was performed on a PBS dispersion of ~10^6^ cells. (**C**) The samples for SR-microFTIR measurements were prepared by separating the cells from the supernatant and depositing them on silicon substrates by physisorption.

The main steps of sample preparation for spectroscopic analysis are sketched in Fig. 1 (for details see Materials and Methods). At first, we present a FTIR analysis of the nanovector, focused on the influence of the AuNPs size on the amplification of the IR signal induced by SEIRA effect. Moving from this, we demonstrate that such a spectroscopic nanoprobe can be internalised in fibroblast cells through medical US and recognised by SR-microFTIR spectroscopy at the single cell level. Moreover, the biological information inferred from the analysis of the SEIRA-amplified spectra of single cells is presented and, in the framework of several nanomedicine studies highlighting the theranostic potential of plasmonic AuNPs^47–49^, compared to the evaluation of the corresponding cytotoxic and genotoxic impact.

### FTIR characterisation of the nanoprobe

The analysis shown in this paragraph is aimed to identify the size of 4ATP-AuNPs probe which better accomplish both efficient US-mediated internalisation within cells and maximisation of the SEIRA signal. Both these requirements are pivotal for the development of an efficient biorecognition tool. To this aim, we assembled different nanovectors as described in Materials and Methods, by conjugating the infrared porter 4ATP with AuNPs, with size ranging between 2 and 20 nm. The upper size of 20 nm was fixed to fulfil the first requirement, according to a previous study^18^. To address the second requirement, we analysed the FTIR response of the differently sized nanovectors, using both traditional (Fig. S2) and SR (Fig. S3) light source, as reported in Section S1 of ESI with the corresponding band assignment (Figs S1 and S2)^28^. From the comparison with the spectrum of bulk IR marker (Fig. S1), an increase of the IR cross-section of the 4ATP, due to the SEIRA effect, occurs, depending on the AuNPs size. In particular, it emerges that the peak at 1595 cm^−1^, corresponding to the asymmetric stretching ν(C = C) of the double bond between carbons of the 4ATP phenyl ring of the bulk sample, experiences both a shift to 1586 cm^−1^ and an intensity enhancement in the nanoprobe samples. Such changes in the spectrum of 4ATP provide evidence that it is effectively conjugated to the AuNPs surface^28^. Notice that a direct comparison of Fig. S2 (by conventional source) versus Fig. S3 (by SR) on the same set of 4ATP-AuNPs shows an evident spectral quality improvement of the SR-microFTIR spectra with respect to the conventional FTIR source. Namely, the vibrational bands associated to 4ATP-AuNPs are neatly identifiable above the noise level even for 2 nm AuNPs.

For each AuNPs size, taking as reference the peak corresponding to the ν(C = C) of the aromatic ring of the SR spectra, we estimated the SEIRA enhancement factor as the ratio between the absorbance measured on the 4ATP-AuNPs samples and that of the bulk 4ATP, each normalised to the number of irradiated molecules in the optical path^30,50^. Such analysis reveals a null gain for the 2 nm AuNPs, a rather low SEIRA enhancement (~4.5) for 5 nm AuNPs, while the enhancement is considerably higher for 10 nm (~137) and 20 nm (~200) AuNPs.

Exhibiting the highest enhancement, we focused on the 4ATP-AuNPs made up of 20 nm AuNPs. A detailed characterisation of the assembling procedure of this nanoprobe was performed by means of UV-Visible spectroscopy, DLS, AFM and FE-SEM, and is reported in Section S2 of ESI, Figs S4, S5, S6 and S7, respectively. From the comparison with non-functionalised AuNPs, the analysis points out a redshift of the absorption peak (from 520.0 ± 0.5 to 522.5 ± 0.5 nm), together with variations in the size (with centre of the distribution shifting from 22 ± 4 to 27 ± 4 nm) and in the ζ-potential (from −34 to −25 mV) of the nanoprobe. These evidences support the spectroscopic analysis, confirming that 4ATP is linked to the AuNPs surface. The negative ζ-potential witnesses a colloidal stabilisation of the nanovector and moreover ensures the electrostatic repulsion with the cell membrane, which is also negatively charged, reducing the risk of alterations of the cell membrane integrity due to non-specific binding^51,52^. In Figs S6 and S7 are reported the optical, AFM (15 μm x 15 μm) and SEM images of the 4ATP-AuNPs deposited on a silicon substrate, which appeared assembled in micrometric aggregates of closely packed AuNPs. Such nanostructured assemblies can sustain plasmonic oscillations in the IR spectral range that we employed in our experiments, thus yielding huge signal enhancement^53,54^.

**Figure 2:**
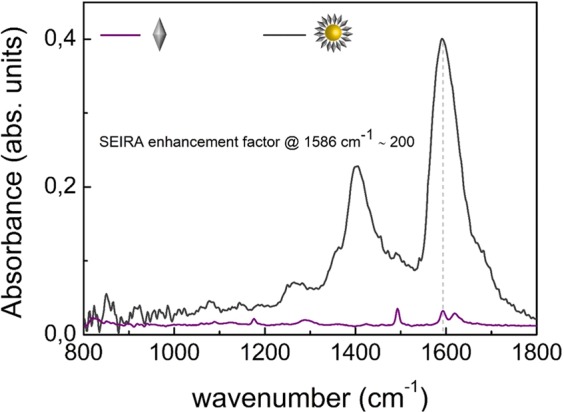
SR-microFTIR spectra of bulk 4ATP (purple line) and of 20 nm 4ATP-AuNPs (grey line) acquired at the Diamond Light Source. The estimated enhancement factor due to the SEIRA effect is of ~200.

The SR-microFTIR spectrum acquired on the same substrate is reported in Fig. 2 in comparison with that of bulk 4ATP. The obtained signal amplification of two orders of magnitude is remarkable, in fact SEIRA enhancement factors reported in literature for AuNPs based substrates can reach values of the orders of 10^2^~10^3^ at best^34,53,55,56^. Hence, 20 nm 4ATP-AuNPs appear as an efficient SEIRA probe for studying the effects of US-mediated drug delivery *in vitro*. Moreover, differently from fluorescent probes employed in other cell signalling strategies, SEIRA probes are not subject to photobleaching which critically affects the reproducibility of the measurements.

Based on these evidences, we carried out the SR-microFTIR spectroscopic study, reported in the following section, aimed to recognise the US mediated internalisation of the 20 nm 4ATP-AuNPs within NIH-3T3 cells.

### SEIRA-based biorecognition of the nanoprobe

**Figure 3:**
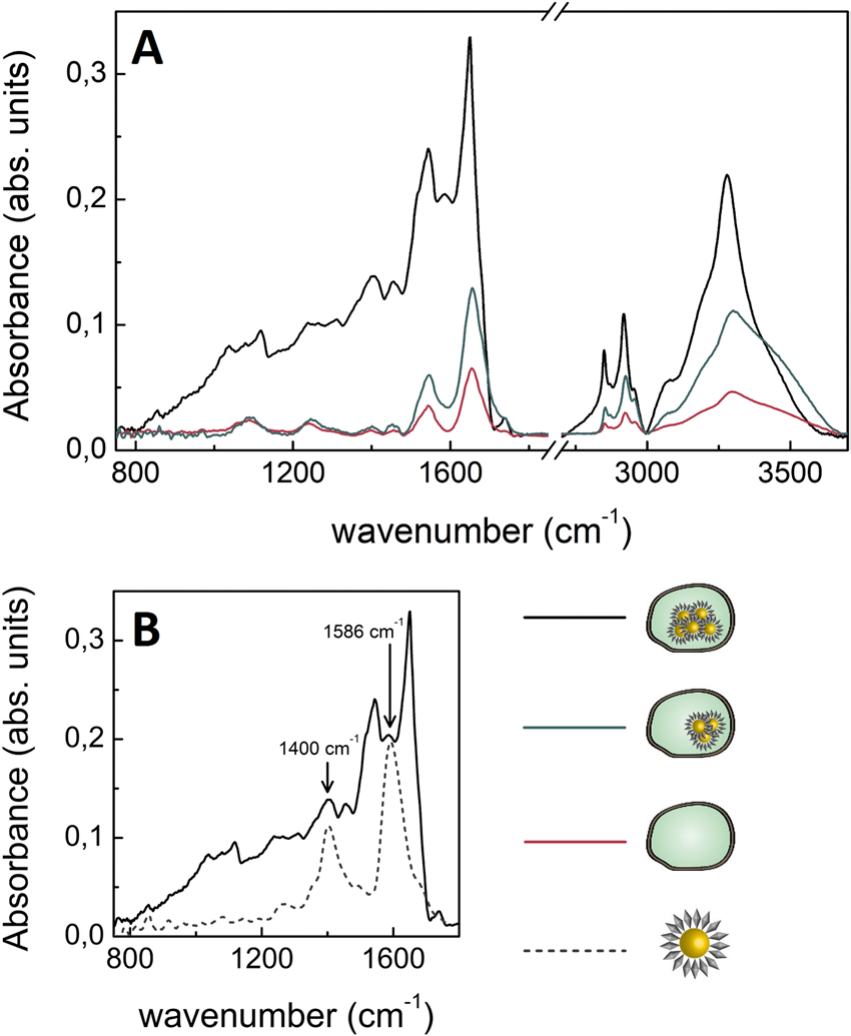
(**A**) SR-microFTIR spectra of non-treated cells (red curve) compared with those of cells treated with 20 nm 4ATP-AuNPs for 15 minutes, which have internalised a different number of nanoprobes by SP, hence showing different modifications of the spectral profile. (**B**) SR-microFTIR representative spectrum of a NIH-3T3 cell irradiated with US (16 mW/cm^2^, 15 minutes) in presence of the 20 nm 4ATP-AuNPs nanoprobe (black line), compared to the SEIRA spectrum of the nanoprobe alone (dashed grey line) is reported; its characteristic peaks at 1400 cm^−1^ and 1586 cm^−1^ can be identified in the spectrum of the treated cell, pointing out the internalisation of the nanoprobe.

NIH-3T3 cells were exposed to US in presence of the 20 nm 4ATP-AuNPs and deposited on silicon substrates (Fig. S8 of ESI) according to Materials and Methods. The nanoprobe internalisation was evaluated by means of SR-microFTIR spectroscopy at the single cell level focusing on the modifications reflecting the presence of the SEIRA nanoprobe coupled with cells. Two types of reproducible spectra can be recognised on the samples, besides the ones unperturbed with respect to the control, non-treated cells. For each case, a representative example acquired on cells treated for 15 minutes is reported in Fig. 3A, in comparison with the control. The first type shows a marked intensity enhancement (up to 10 fold) and evident changes in the spectral shape, mainly in the protein amides region (Amide I at 1655 cm^−1^ and Amide II at 1545 cm^−1^, according to the band assignment of ref.^22^), together with the appearance of two peaks, at 1400 cm^−1^ and 1586 cm^−1^. In the second type, the intensity enhancement is less evident and it is not possible to recognise the two additional peaks. Nevertheless, even in this case some modifications of the spectral shape are observed, mainly in the amides region.

In Fig. 3B a representative spectrum of the first type, showing the markedly amplified intensity and the two additional peaks, is compared to that of the nanocarrier alone. Since the SEIRA intensity enhancement is induced by AuNPs clusters large enough to achieve a plasmonic resonance in the IR spectral region, the amplification of the spectrum reported in Fig. 3 demonstrates that a sufficient number of nanoprobes have been internalised by cells. The two additional peaks can be clearly ascribed to the presence of 4ATP-AuNPs^28^, as highlighted by the superimposition of their spectral profile. This provides a further evidence of the nanoprobe internalisation and states that the molecular probe is still anchored to the gold surface, thus pointing out the stability of the carrier. It should also be noted that the increased intensity in both C-H and N-H stretching spectral regions, at 2800~3000 cm^−1^ and 3200~3500 cm^−1^ respectively (Fig. 3A), is a strong indication of the coupling of 4ATP-AuNPs probe and cells, which occurred to sonicated samples only.

The internalisation efficiency was also evaluated by the percent ratio of the number of amplified spectra in which it is possible to recognise the peaks associated to the 4ATP marker, out of a total of 20 screened cells. We obtained a 10% uptake on samples treated for 15 minutes and 30% on those treated for 30 minutes, rather low if compared with the values previously reported on cells treated in the same conditions using dextran with Stokes diameter of ~12 nm (50% uptake upon 15 minutes treatment)^18^. A more coherent estimation is obtained if also the second type of cell spectra, in which the signal amplification is lower, is taken into account. This yields uptake efficiencies of 40% and 60% for exposure times of 15 and 30 minutes, respectively.

Another aspect to consider is the cell sonication procedure. Indeed, as described in Materials and Methods, cells for SR-microFTIR analysis were treated in suspension, and then laid onto the mid-infrared transparent substrate (silicon wafer). Although usually the SP treatments are made on cells growing in adhesion conditions, the specific case our protocol has some advantages. Indeed, it allows work with high cell concentration in small volumes of treatment in order to limit the dilution of the nanoprobe, it avoids some possible artefacts due to the interaction of cells with silicon surface, it maximises the exposed surface of cell plasma membrane, and it avoids membrane perturbation by post SP biochemical treatments (e.g., cells detachment procedures).

In summary, the SEIRA based detection of 4ATP-AuNPs inside cells allowed us to characterise the SP-mediated internalisation. Our results appear particularly relevant in terms of both uptake efficiency and sensitivity at the single cell level considering that the SEIRA signal enhancement depends on the number of 4ATP-AuNPs internalised per cell and that the initial concentration of the AuNPs dispersion is extremely low (~ nM). However, by adopting a strategy to concentrate 4ATP-AuNPs at the cell-medium interface undergoing US, our results may be, in principle, further improved. As mentioned in the Introduction, the SEIRA methodology using SR-microFTIR can offer the advantages of ease of preparation and accuracy compared with fluorescence and electronic microscopies (e.g., TEM) where the sample preparations are quite laborious, prone to artefacts, invasive and the biochemical information is difficult to be achieved^57^. In addition, the detection sensitivity of AuNPs by microfluorescence techniques is dramatically reduced because of the well-known phenomenon of fluorescence quenching of dye molecules near the gold surface.

For completeness, it is important to stress here that the FTIR evidences induced by US and AuNPs combined treatment were compared also with those provided by US or AuNPs separately. Although neither additional peaks nor spectral amplifications due to SEIRA phenomenon were detected with respect to the untreated control samples, a detailed analysis to understand contributions of individual stimuli to the biochemical structure is reported in in the following section.

The lack of SEIRA nanoprobe perturbation in the FTIR profile of non-sonoporated cells clearly indicates that for an efficient internalisation of 4ATP-NP probe the membrane SP is required. On this line, it has been recently demonstrated that the 4ATP-AuNPs used here can be bio-functionalised further with molecules that provide specific affinity to receptors expressed by cells in a particular stage of their life cycle, or which are in particular pathological conditions^40^, to be selectively recognised on the cell plasma membrane. This would mean that our nanoprobe can be suitably improved to be employed for triggering combined chemical and US cell targeting and spectral imaging. In this direction, a combined analysis of the changes in the FTIR spectrum of native NIH-3T3 cells together with cytotoxic assays of the treatments is carried out in in the next section.

### Analysis of the biological effects induced by the nanoprobe internalisation

In the previous section we demonstrated that, by exploiting transient membrane SP, it is possible to efficiently internalise our 4ATP-AuNPs nanoprobes within NIH-3T3 cells and that the SEIRA amplification of the cell spectra allows the biorecognition of the nanoprobe at the single cell level, with extremely high sensitivity. Proceeding this, we studied the biological side effects related to the nanoprobe internalisation. To this aim we evaluated the modifications occurring in the SR-microFTIR spectra of cells treated with US in presence of 4ATP-AuNPs for 15 and 30 minutes and supported the spectral analysis with cytotoxicity and genotoxicity assays.

We focused our analysis on a significant portion of treated cells exhibiting the spectra we named herein “of second type”, where the additional peaks attributed to the nanovector do not appear and the intensity enhancement is not pronounced. A rather low number of internalised 4ATP-AuNPs is in fact expected in the corresponding cells, thus spectral distortions associated to the SEIRA effect^58,59^ are minimised, as well as possible biological responses due to the crowding of the nanoprobes in the cytosol. Thus any observed effect can be reasonably attributed to cell-nanoprobe biochemical interactions. Specifically, we analysed the fingerprint region (between 800 and 1800 cm^−1^, Fig. S9A, Section S3 of ESI), where all the most significant bands associated with the molecular vibrations of proteins and nucleic acids are located, and the lipid region (between 2800 and 3000 cm^−1^, Fig. S9B, Section S3 of ESI)^60^. The spectra of treated samples show evident shift and broadening of the Amide I band, particularly marked in cells that were treated for longer time. Such modifications are discernible on several spectra (Fig. S10), in which shift and broadening of the Amide II can be also recognised. Similar effects are moreover observed in some cells sonicated in presence of 5 nm 4ATP-AuNPs, where the SEIRA effect was negligible (Fig. S11). In the sample treated for 30 minutes with 20 nm 4ATP-AuNPs a concomitant slight decrease occurs in the intensity of the bands at 1086 and 1238 cm^−1^_,_ corresponding to the DNA phosphate stretching modes. Such decrease is indicative of chromatin condensation^61^. According to the literature, all the observed spectral modifications can be attributed to the onset of apoptotic processes in the treated cells^61,62^. In addition, weak variations are observed in the intensity of the CH_2_ and CH_3_ stretching modes at 2851 and 2958 cm^−1^, respectively, which might be related to structural alterations in the cell membrane, also associated with apoptotic events^17,63^.

**Figure 4:**
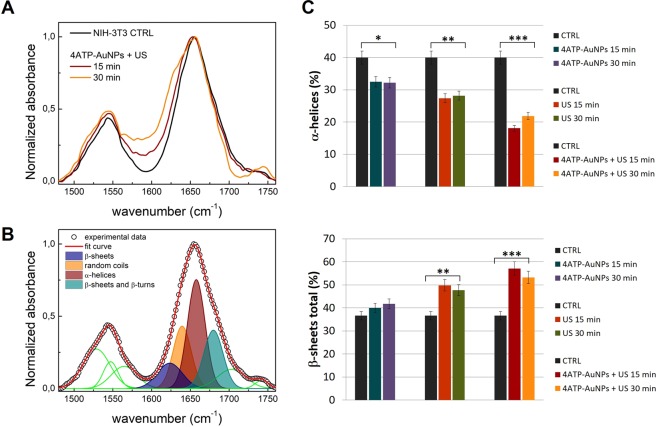
(**A**) Amide region of the SR-microFTIR spectra of the samples treated with both US and 4ATP-AuNPs for 15 (red line) and 30 minutes (orange line) in comparison with the non-treated control sample (black line); the spectra are normalised to the absorbance of the Amide I band. (**B**) Representative spectral deconvolution by Gaussian fitting procedure; the spectral components associated to the protein secondary structure are highlighted by filled colour areas (the other spectral components are represented in green). (**C**) Histograms representing the weight of α-helices (top) and β-sheets (bottom) spectral components in the Amide I band for each sample analysed; each weight was determined as the percent area with respect to the total area of the band. The β-sheets percentage accounts for both the β-structure contributions (1624 cm^−1^ and 1678 cm^-1^). Columns and bars represent the average and standard deviation values obtained on at least three independent experiments. *p < 0.05, **p < 0.01 and ***p < 0.001.

With the purpose to deepen the analysis of the biological effects induced by the combined treatment with US and 4 ATP-AUNPs, we focused on the amides region, between 1450 and 1800 cm^−1^, where the most evident spectral changes are located (Fig. 4A). We therefore compared the spectra of NIH-3T3 cells treated for 15 and 30 minutes with those of corresponding samples exposed to US or 4ATP-AuNPs separately, in order to disentangle the different contributions. We analysed each spectrum by a Gaussian curves deconvolution, as shown in the representative graph of Fig. 4B. Four major components were assigned to the Amide I band and reported in Table 1.

**Table 1:** Peak assignment of the spectral components resulting from the Amide I band Gaussian deconvolution, according to refs^60,72^.

Peak centre (cm^−1^)	Assignment
1624	β-sheets
1639	random coils
1658	α-helices
1678	β-sheets and β-turns

The percent area of each component, with respect to the total area of the band, was determined for all the samples and reported in the histograms of Fig. S12 of ESI, in comparison with those of the control non-treated cells. The histograms relative to the α-helices and to the total weight of the β-structures (sum of the contributions of the peaks at 1624 cm^−1^ and 1678 cm^−1^) are shown in Fig. 4C.

Samples treated with the nanoprobe only (non-sonicated) do not show substantial spectral variations with respect to the control sample. Only a slight decrease in the α-helices components is observed, along with an increase in the β-sheets percentage depending on the treatment time. Concerning the exposure to US (without the nanoprobe), the spectral changes are more pronounced. In particular, the weight of the β-sheets components rises up to 50% from the 37% of the control. Such US induced modifications of the protein secondary structure are coherent with those previously reported^22^. The samples treated with both US and 4ATP-AuNPs show a dramatic drop of the α-helices components, associated to an increase of the β-sheets contributions.

The weight of random coils does not show significant changes for all the examined samples (Fig. S12). Therefore the observed structural changes may be symptomatic of early stages of the apoptotic cellular response. In this regard, Buriankova *et al*^64^. reported that photo-dynamically treated human glioma cells suffered a passage in the early stage of apoptosis which has been correlated to a shift of the infrared Amide I band of 26 cm^−1^, attributable to conformational transition of the secondary structure of proteins from α-helix to β-sheet.

Bearing all these considerations in mind, we suppose that some apoptosis events may occur starting from 15 minutes of US-nanoprobe combined treatments. If confirmed, our hypothesis might have some relevance in therapy scenarios where US can be highly focused in specific points of the body to trigger a controlled cell death. In this framework, given the possible effects in the cellular microenvironment caused by our nanosystem, it would be appropriate to investigate the eventual presence of a threshold of toxicological concern.

**Figure 5:**
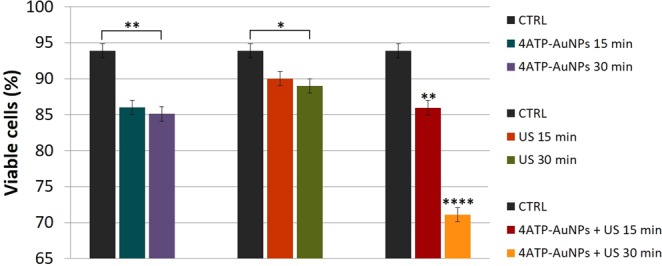
Cell viability determined upon the different treatments by flow cytometry Annexin V and PI combined assay as the percent number of viable cells with respect to the total population of samples analysed. Viability of non-treated control samples (black column) is the same repeated three times. Columns and bars represent the average and standard deviation values obtained on at least three independent experiments. *p < 0.05, **p < 0.01, ***p < 0.001 and ****p < 0.0001.

To shed light on the biological effects revealed by the SR-microFTIR analysis, we carried out a flow cytometry Annexin V and PI combined assay, together with a cleaved caspase-3 immunofluorescence staining. In this way it was possible to monitor the eventual onset of apoptosis and necrosis processes. FITC-labelled Annexin V binds the phosphatidylserine translocated to the outer cell membrane layer during apoptosis, while PI is internalised within cells only upon membrane damage, occurring during late apoptosis and necrosis processes. The combined assay, performed in triplicate according to Material and Methods, allows therefore to distinguish between the different cell death pathways. The results, obtained on samples underwent the different treatments, are reported in Fig. S13 of ESI. In Fig. 5 the cell viability is reported as the percentage of viable cells with respect to the total population analysed.

The analysis points out a slight loss of viability when cells undergo US-nanoprobe combined treatment for 15 minutes. Marked effects occur for the 30 minutes treatments. This is consistent with the results of the previous section on the 4ATP-AuNPs uptake. The cells undergoing US or 4ATP-AuNPs individual treatments show a weak cytotoxic impact independently from incubation time, with a slightly lower impact on mortality of US. In accordance with the SR-microFTIR, we can thus deduce that it is the US-nanoprobe combined effect that activates some cell death pathway. On the other hand, it has been reported that the US irradiation of nanoparticles can enhance cavitation effects. It is therefore possible that this phenomenon also contributes to the observed increase of the cytotoxic response in samples that underwent combined US-nanoprobe treatments with respect to those treated by US or 4ATP-AuNPs separately^5^.

In agreement with reports in the literature^18^, our results also indicate that after 30 minutes treatment, US pressure waves begins to cause irreversible damage to the cell membranes. Fig. S13 of ESI also suggests that the US or 4ATP-AuNPs individual treatments provide an analogous effect in triggering the first events that then lead to apoptosis; likewise, combined US-nanoprobe treatments (15 and 30 minutes) give an almost identical percentage of early apoptotic cells and show a small increase of apoptosis compared to the individual treatments at the same times. The flow cytometry results confirmed the hypothesis based on our SR-microFTIR analysis and it would be interesting to investigate further if the latter will result in a more sensitive tool than flow cytometry, managing to distinguish early apoptotic cells also between the different treatments.

It is worth noting that overall treatments affect slightly the cell viability mostly due to necrosis (Fig. S13). More interesting, the combined US and nanoprobe treatments also induce a quite significant percentage of apoptotic events. Specifically, early apoptotic cells which appeared in response to the 15 minutes combined treatment became late apoptotic after 30 minutes, allowing us to conclude that the time of US-nanoprobe combined exposure is crucial to trigger irreversible apoptotic events on NIH-3T3 cells.

The actual occurrence of apoptosis was also investigated by cleaved caspase-3 immunofluorescence analysis, since caspase-3 is the final effector of apoptosis after being activated through hydrolytic cleavage by caspase-8 (extrinsic pathway) or caspase-9 (intrinsic pathway) (Fig. S14 and Fig. S15 of ESI). Non-treated cells used as controls all tested negative for cleaved caspase-3, while our positive control of apoptosis, represented by staurosporine treated cells, tested 60% positive. At the same time, 2% of the cells exposed to US, independently from the combined treatment with 4ATP-AuNPs, showed positive for cleaved caspase-3, demonstrating an activation of the apoptotic process. Also the treatment with 4ATP-AuNPs alone without US exposure induced 1~2% of apoptosis, evidenced by cleaved caspase-3 positivity. Obviously, the percentages obtained by immunofluorescence are indicative only, being a semi-quantitative test, however the results are comparable with the flow cytometry data, except for the case where the sample underwent US-nanoprobe combined treatment for 30 minutes.

**Figure 6:**
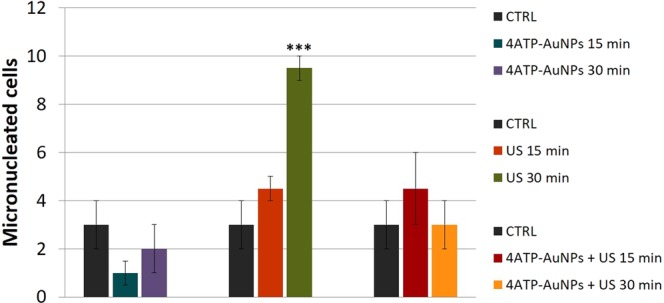
Average number of micronucleated cells (error bars represent the standard deviation), determined upon the different treatments by the CBMN assay. The values are obtained analysing a population of 500 cells for each sample. ***p < 0.001.

A CBMN assay was performed according to Section 2.8 to evaluate potential genomic instability at chromosomal level and the frequencies of micronucleated cells on the samples which underwent the different treatments. The results of the analysis are reported in Fig. 6 in terms of number of micronucleated cells.

The results show, in a seemingly surprising way that are the samples treated with US only to show a slight increase in the number of micronucleated cells, which becomes significant when cells are exposed to US for 30 minutes (in Fig. 6 compare violet and green histogram columns, respectively, with the control one in dark grey). Contrarily, the US-nanoprobe combined treatments correspond to comparatively lower micronuclei frequency which does not differ significantly from those measured in the control samples. According to the cytotoxicity analyses, after the US-nanoprobe combined treatments, late apoptotic cells appeared, and, in this respect, both events of cell death via apoptosis and decrease of binucleated cells may be related. Moreover, the intensity decrease in the DNA phosphate stretching infrared modes observed on the same sample (Fig. S9A), indicative of chromatin condensation, is correlated to apoptotic cell death^61^.

The potential genotoxic impact of medical US has been only slightly investigated so far^19,21,65–67^. The positive results obtained for 30 minutes treatment under subcavitation conditions support the hypothesis that in addition to the effect of transient SP of the plasma membrane^23^, some probable effect on the genomic integrity might be expected.

Any chromosomal damage related to the phenomenon of micronucleation induced by US could result from a direct mechanical stress on the nucleus or indirectly transmitted to it^68^. In this regard, some speculations can be made considering our specific irradiation conditions. Indeed, although the nucleus is the most rigid cellular organelle (i.e. 2~10 times more rigid than the surrounding cytoskeleton), the instantaneous pressure values involved herein (10~60 kPa), while being too low to directly induce reactive oxidative species, were higher than the “threshold stress” (0.1~10 kPa) needed to induce nuclear deformation^69^. According to the literature, mechanical input on the nuclear membrane barriers^69^ and at the level of the mitotic spindle structure of the cell^70,71^ is one of the mechanisms through which the present effect becomes manifest (i.e. post-mitotic micronuclei from lagging chromatids or chromatin bridges between anaphase chromosomes).

## Conclusions

In this work we investigated the possibility to recognise SEIRA-detectable 4ATP-AuNPs internalised within the NIH-3T3 murine fibroblast cell model by transient membrane SP. The optimal conditions in terms of energy and exposure time to successfully trigger the uptake of the nanoprobes inside cells, with a minimal impact on their viability, were determined on the basis of previous investigations^18^. The coupling between the high sensitivity of SR-microFTIR spectroscopy and the considerable signal enhancement provided by SEIRA effect enabled us to recognise the spectral fingerprint of the nanoprobe at the single cell level, providing convincing evidence of a successful internalisation.

The high-quality of the acquired spectra allowed recognition of biological effects related to a condition of early apoptosis, resulting from the interaction of the cell with the nanoprobe as well as with US. These aspects have been thoroughly investigated by cytotoxicity and genotoxicity assays. Flow cytometry and immunofluorescence combined analysis indicated that in all treatments a slight decrease of vitality occurs. Moreover, the CBMN assay of the genomic instability pointed out a slight increase in the number of micronucleated cells, which becomes less significant in the case of the US-nanoprobe combined treatment for 30 minutes, corresponding to the onset of late apoptosis processes. These results intriguingly match with the FTIR analysis, indicating that SR-microFTIR can be useful for early screening in support of common toxicological techniques.

Developing methodologies of delivering and detecting nanovectors able to modify the biodistribution, tissue uptake and pharmacokinetics of therapeutic agents, is considered of great importance in biomedical research. In this respect we are confident that the progresses in ultrasonic nanoparticle delivery and optical detection combined strategies herein reported can provide novel insights of theranostics relevance.

## Supplementary information

Supplementary Information
