# Supplementary material for: Ultrasound delivery of Surface Enhanced InfraRed Absorption active gold-nanoprobes into fibroblast cells: a biological study via Synchrotron-based InfraRed microanalysis at single cell level
Source: Sci Rep. 2019 Aug 14;9:11845. doi: 10.1038/s41598-019-48292-0 (PMC6694135; doi:10.1038/s41598-019-48292-0)
Supplement: Supplementary file 1 — Supplementary Information [file 41598_2019_48292_MOESM1_ESM.pdf]

ELECTRONIC SUPPLEMENTARY INFORMATION

Ultrasound delivery of Surface Enhanced InfraRed Absorption active gold-nanoprobes into fibroblast cells: a biological study via Synchrotron-based InfraRed microanalysis at single cell level

F. Domenici[1*], A. Capocefalo[2], F. Brasili[1,2], A. Bedini[3], C. Giliberti[3], R. Palomba[3], I. Silvestri[4], S. Scarpa[5], S. Morrone[5], G. Paradossi[1], M.D. Frogley[6], G. Cinque[6]

[1] Dipartimento di Scienze e Tecnologie Chimiche, Università degli Studi di Roma "Tor Vergata", Rome, Italy

[2] Dipartimento di Fisica, Università degli Studi di Roma "Sapienza", Rome, Italy

[3] Dipartimento Innovazioni Tecnologiche e Sicurezza degli Impianti, Prodotti e Insediamenti Antropici (DIT), INAIL, Monteporzio Catone, Rome, Italy

[4] Dipartimento di Medicina Molecolare, Università degli Studi di Roma "Sapienza", Rome, Italy

[5] Dipartimento di Medicina Sperimentale, Università degli Studi di Roma "Sapienza", Rome, Italy

[6] MIRIAM beamline B22, Diamond Light Source, Harwell Campus, Chilton-Didcot OX11 0DE, U.K.

* Corresponding Author; e-mail: fabio.domenici@uniroma2.it

S1. FTIR analysis of the different nanoprobes

In this section are reported the preliminary FTIR and SR-microFTIR measurements that allowed us to determine which size of the tested AuNPs (2, 5, 10 and 20 nm) provides the strongest SEIRA amplification. The FTIR spectra of the bulk 4ATP and those of the 4ATP-AuNPs nanovectors with different size, obtained employing a traditional IR source, are reported in Figure S1 and Figure S2 with the corresponding band assignment. The spectra acquired with the SR light source are reported in Figure S3.

It is possible to notice that, upon the conjugation with the AuNPs, the peak relative to the asymmetric stretching of the double bond C=C of the phenyl ring of the 4ATP shifts from 1595 to 1586 cm^{-1}. This peak was taken as reference in the calculation of the SEIRA enhancement factors. In addition, a direct comparison of Figure S2 versus Figure S3 on the same set of 4ATP-AuNPs shows an evident spectral quality improvement of the SR-microFTIR spectra with respect to the conventional FTIR source. Namely, the vibrational bands are neatly identifiable above the noise level even for 2 nm AuNPs.

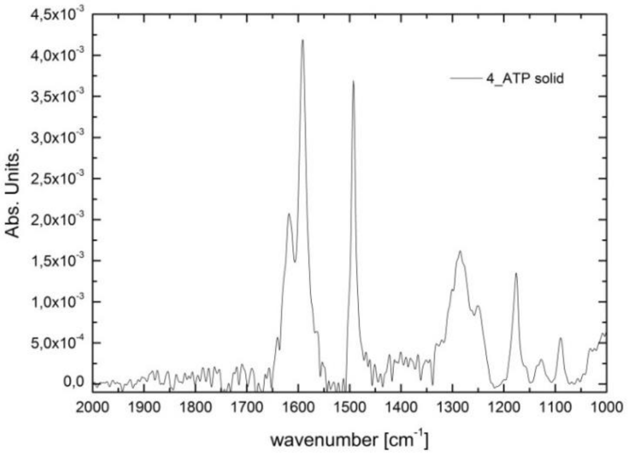

Assignment	Wavenumber (cm^{-1})
***γ*CC + *γ*CCC**	1010 w
***ν*CS**	1090 m
***δ*CH**	1176 w
***ν*CH**	1284 m
***ν*CC + *δ*CH**	1423 w
***ν*CC + *δ*CH**	1493 s
***ν*CC**	1595 s
***δ*NH**	1620 s

Figure S1: FTIR spectrum of bulk 4ATP measured by traditional IR source. The band assignment, in accordance with ref. [28], is reported in the table on the right. Greek letters indicate the vibrational modes: *ν*, stretching; *δ* and *γ*, bending. The letters in the second column indicate the relative intensities of the bands: s, strong; m, medium; w, weak. The row highlighted in light blue identifies the band νCC, which shifts upon conjugation with AuNPs.

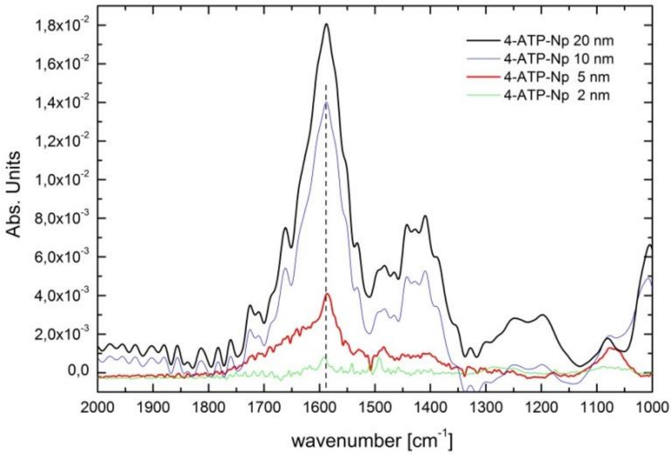

Assignment	Wavenumber (cm^{-1})
***γ*CC + *γ*CCC**	1008 w
***ν*CS**	1079 w
***δ*CH**	1176 w
***ν*CH**	1280 m
***ν*CC+ *δ*CH**	1400 m
***ν*CC+ *δ*CH**	1488 m
***ν*CC**	1586 s
***δ*NH**	1629 m

Figure S2: SEIRA spectra measured by traditional IR source on the 4ATP-AuNPs nanoprobes prepared using AuNPs of different size. The band assignment, in accordance with ref. [28], is reported in the table on the right. Greek letters indicate the vibrational modes: *ν*, stretching; *δ* and *γ*, bending. The letters in the second column indicate the relative intensities of the bands: s, strong; m, medium; w, weak. The dotted line in the graph and the row highlighted in light blue in the table identify the band νCC, shifted to 1586 cm^{-1}, which allows us to recognise the linker conjugated to AuNPs and to identify the highest enhancement due to SEIRA effect.

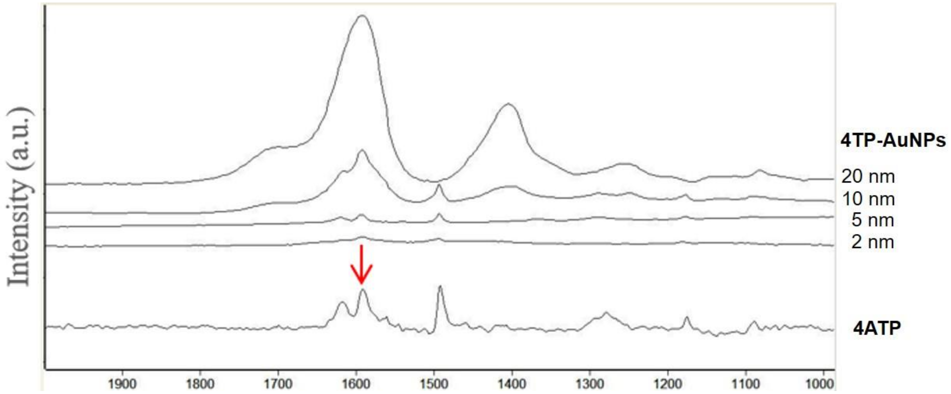

Figure S3: SR-microFTIR spectra of bulk 4ATP and of 4ATP-AuNPs prepared using AuNPs with different size. From the bottom: 4ATP, 2 nm 4ATP-AuNPs, 5 nm 4ATP-AuNPs, 10 nm 4ATP-AuNPs, 20 nm 4ATP-AuNPs. The red arrow indicates the 4ATP vibrational mode νCC.

S2. Characterization of the 20 nm 4ATP-AuNPs nanoprobe

The different steps of the preparation procedure of the 20 nm 4ATP-AuNPs nanoprobe were monitored by means of UV-Visible absorption spectroscopy, Dynamic Light Scattering and Atomic Force Microscopy (AFM). UV-Visible measurements (Figure S4) point out the redshift of the surface plasmon resonance of the AuNPs upon functionalization with 4ATP, allowing to confirm the coverage of the gold surface. Dynamic Light Scattering measurements (Figure S5) provided information on the size and surface charge of the water dispersed 4ATP-AuNPs in terms of hydrodynamic radius and ζ-potential. AFM (Figure S6) and FE-SEM (Figure S7) morphological information on the nanoprobes were obtained on the 20 nm 4ATP-AuNPs deposited on a silicon substrate in comparison to the bare AuNPS.

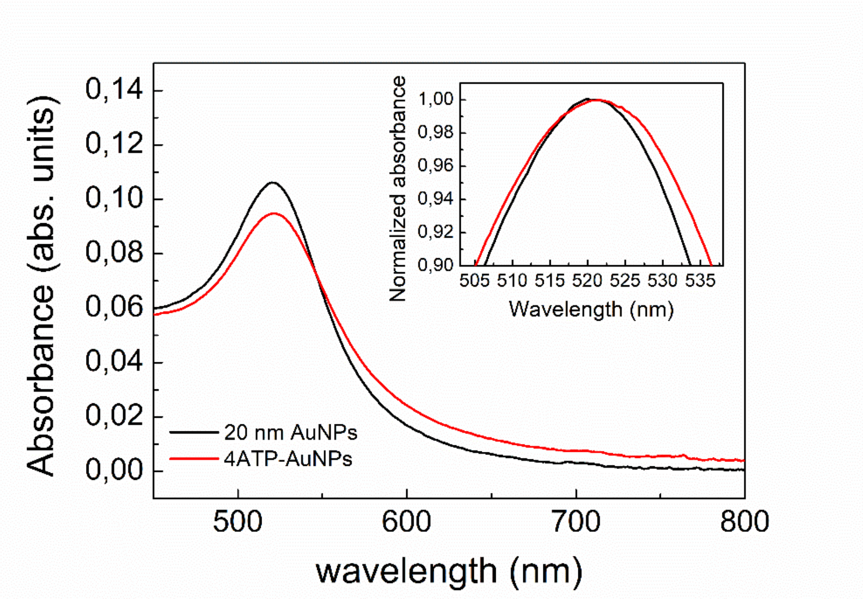

Figure S4: UV-Visible absorption spectra of the 20 nm AuNPs before (black) and after the conjugation with 4ATP (red curve). In the inset is reported a zoom of the spectra normalised to the maximum absorbance value, highlighting the red-shift of the AuNPs absorption peak upon the functionalization.

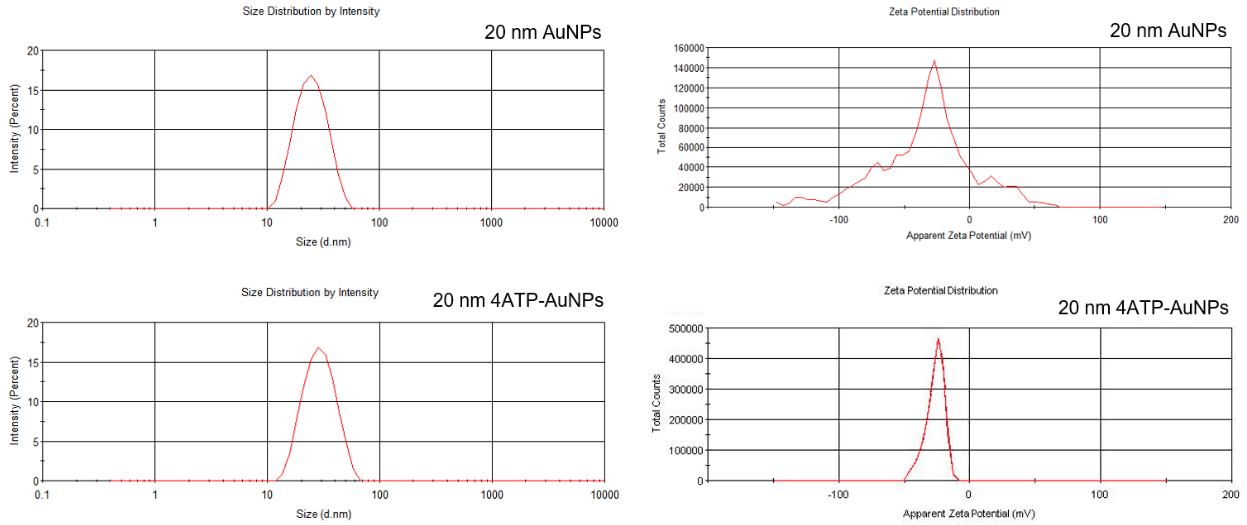

Figure S5: Size and ζ-potential distributions of the 20 nm AuNPs before (left) and after the conjugation with 4ATP (right). 20 nm AuNPs shows an average size of 22±4 nm which increases to 27±4 nm after the conjugation with 4ATP. ζ-potential of 20 nm AuNPs shows a peak at -34.2 mV indicating an electronegative coverage of the nanocolloids. The complex 4ATP-AuNPs presents a peak at - 24.9 mV.

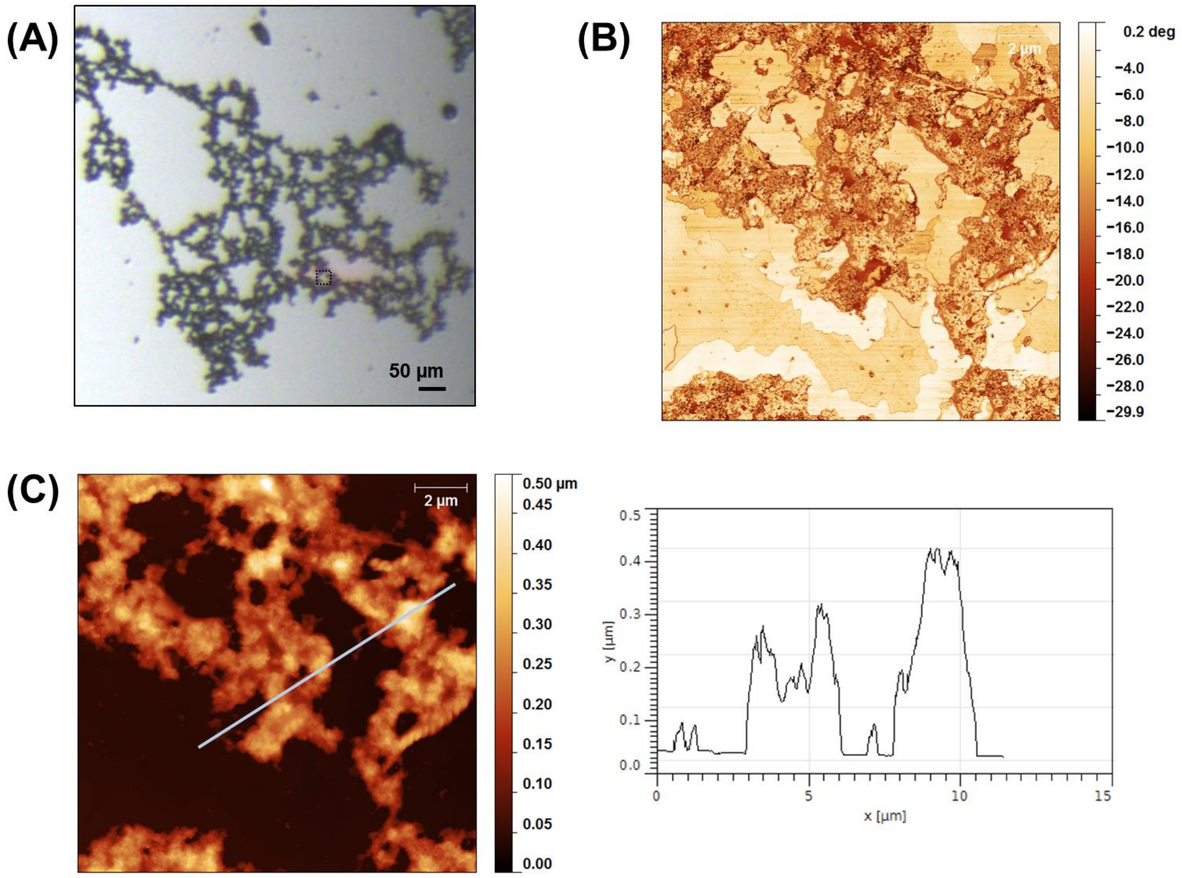

Figure S6: (A) Optical microscopy image of the 20 nm 4ATP-AuNPs colloids evaporated on a silicon substrate. (B) AFM phase image (B) and topography analysis (C) of the deposition, with the white line indicating the section of the profile showed in the last image.

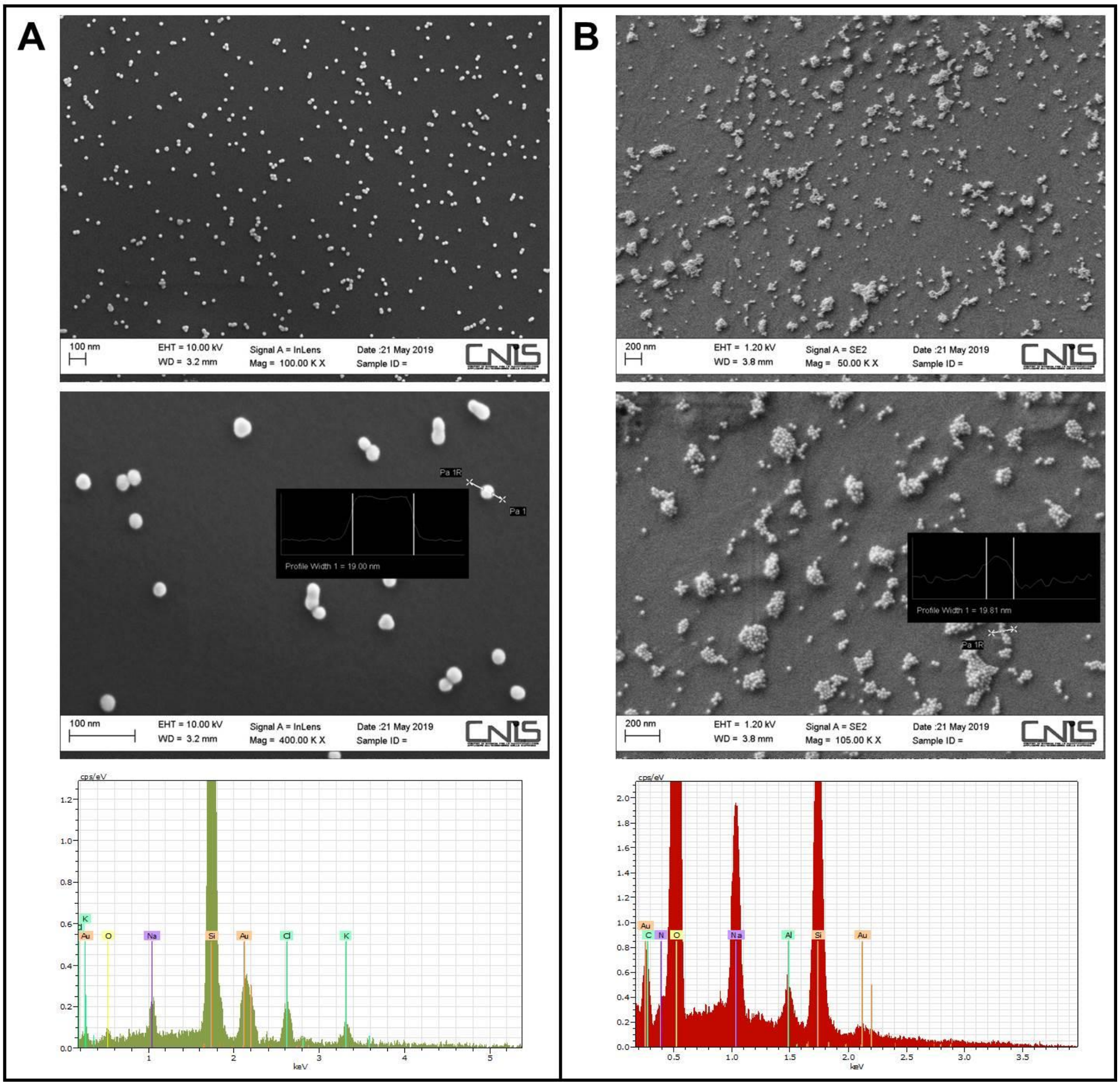

Figure S7: FE-SEM analysis of 20 nm AuNPs (A) and 4ATP-AuNPs (B) colloids evaporated on a silicon substrate, together with the corresponding microanalysis.

S3. SR-microFTIR investigation on single cells

In this section is reported the SR-microFTIR analysis performed on the NIH-3T3 line aimed to assess at the single cell level the transient sonoporation mediated internalization of our 20 nm 4ATP-AuNPs nanoprobe and to evaluate the associated biological effects. Representative images of the substrates employed for the measurements are reported in Figure S8, where the 15×15 µm slits of the microbeam are highlighted. Depending on the number and crowding of internalised nanovectors, two types of spectra can be recognised in the treated cells, as reported in Figure 3 of the main text, in comparison with the spectrum of a control non-treated cell.

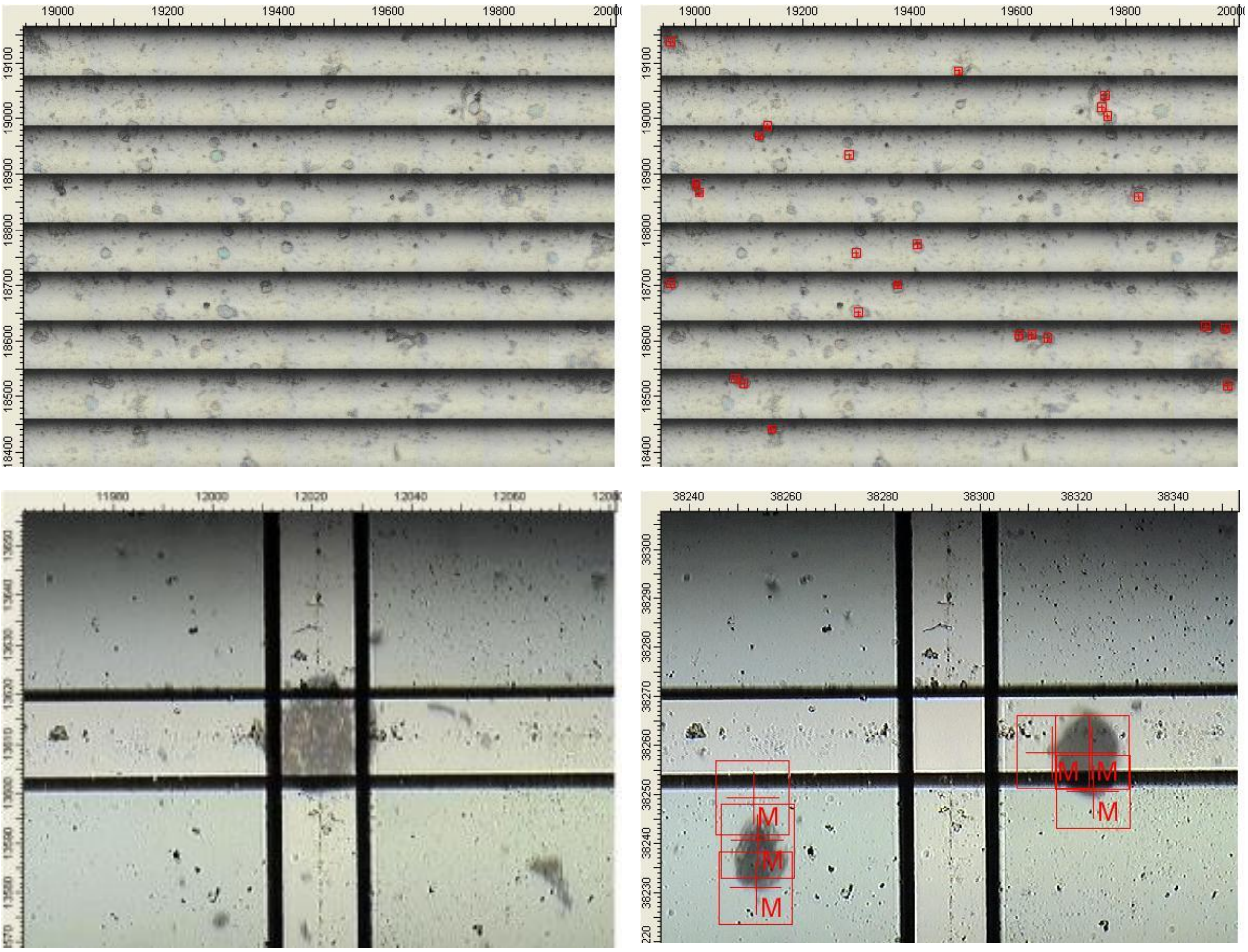

Figure S8: On top, a 2-D cell map selected by FTIR microscope Hyperion 3000, and analysed cell by cell through a microbeam of 15×15 µm (right); On bottom, a magnification on single fibroblasts selected, and Marked (right) for the SR microFTIR analysis. NIH-3T3 cells were laid onto the silicon surface upon treatment and measured according to the Materials and Methods section of the main text, respectively.

The spectral modifications, associated to the biological effects induced in the treated cells, occur both in the fingerprint (proteins and nucleic acids) and in the high frequencies (membrane lipids) spectral regions, as reported in Figure S9. In particular, the Amide I and Amide II show the most evident spectral changes as highlighted by the zoom of Figure S10. Similar effects were observed also in cells treated with 5 nm 4ATP-AuNPs, whose SEIRA enhancement is negligible, as showed in Figure S11. The spectral deconvolution analysis of the amides region, performed by Gaussian band fitting and reported in Figure S12, pointed out an increase in the weight of the β-sheets component along with a decrease in that of α-helices, depending on the different treatments.

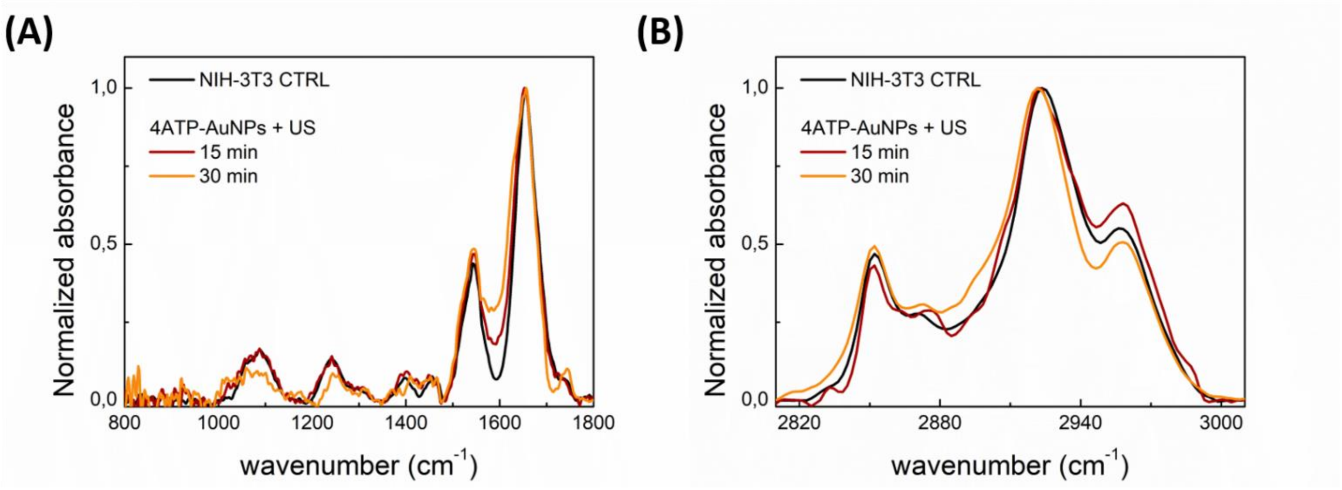

Figure S9. Fingerprint (A) and high frequencies (B) SR-microFTIR spectra of samples underwent US-nanoprobe combined treatments for 15 (red curve) and 30 (orange curve) minutes, in comparison with the non-treated control sample (black curve).

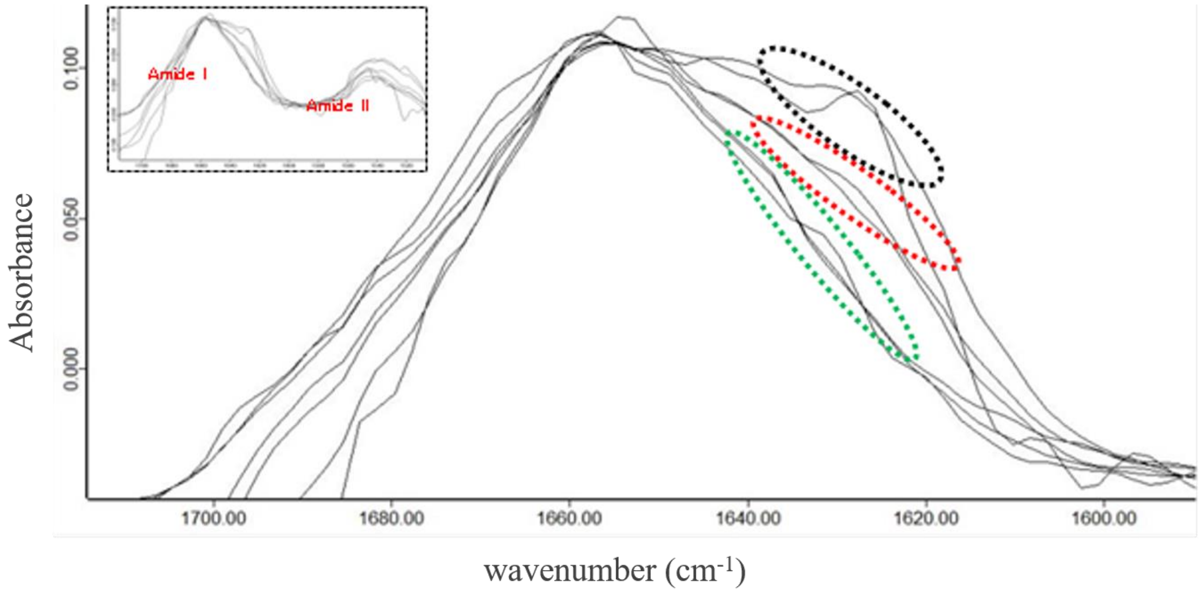

Figure S10: Zoom on the Amide I band of NIH-3T3 samples underwent US-nanoprobe combined treatment for 30 minutes. The broadening and the shift of the band to lower frequencies, due to the increase in the β-sheets contribution are highlighted.

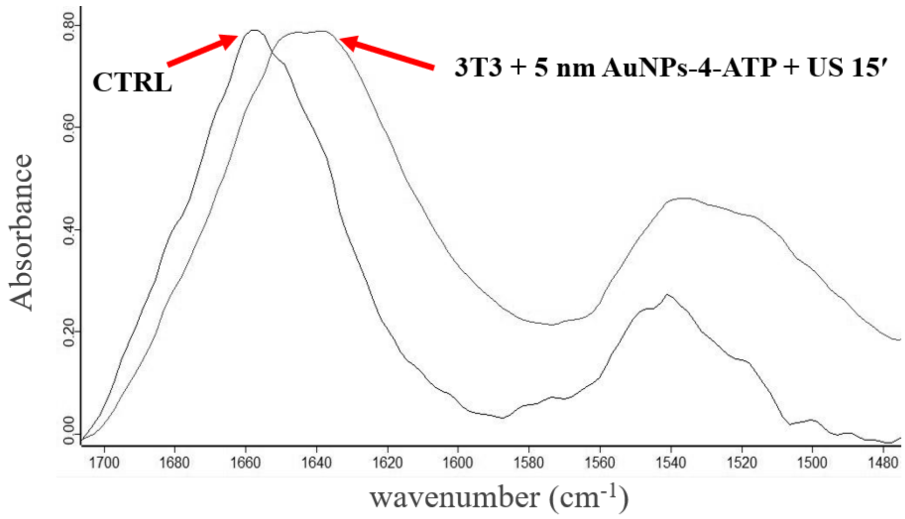

Figure S11: Amide bands of a NIH-3T3 sample treated for 15 minutes with 5 nm 4ATP-AuNPs and US, compared with the control non-treated sample. The redshift and a broadening of Amide I are coherent with those observed on cells treated with the 20 nm nanoprobe.

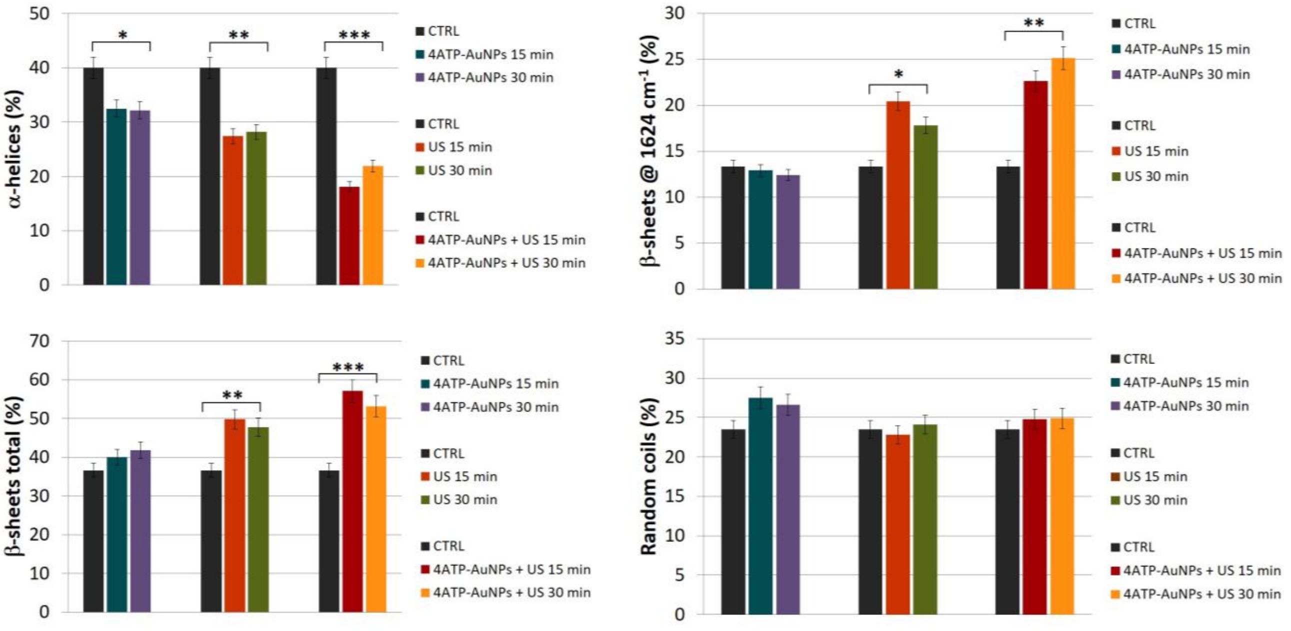

Figure S12: Histograms representing the percentage of secondary structure of the cell protein composition extrapolated from the Amide I spectral decomposition. *p<0.05, **p<0.01 and ***p<0.001.

S4. Cytotoxicity assays

In this section are reported the assays performed to reveal the cytotoxic response of NIH-3T3 cells upon the transient sonoporation mediated internalization of our 20 nm 4ATP-AuNPs. All the treatments were performed at the same nanoprobe concentration (dilution by a factor 4 of the NPs stock dispersion) and US doses used for sonoporation experiments. The results of the flow cytometry AnnexinV/PI combined assay, distinguishing the different death pathways, are reported in Figure S13. For cleaved caspase-3 expression assay, cell growth chambers known as chamber slides (Nunc, Naperville, IL, U.S.A.) each containing 8 wells were used. Each well was filled with a volume of 500 µl of DMEM grow medium containing 5000 NIH-3T3 cells. A scheme of a cell filled chamber slide together with microscopy images representative of the NIH-3T3 cells undergoing US and NPs treatments are shown in Figure S14. Immunofluorescence results obtained by Olympus BX52 fluorescence microscope are reported in Figure S15, where red spots denote the positive expression of cleaved caspase-3.

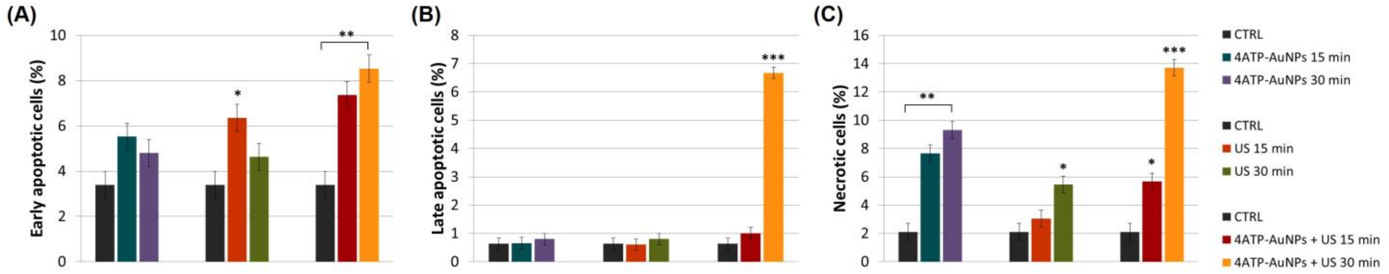

Figure S13: Flow cytometry analysis of the NIH-3T3 cells performed by the AnnexinV/PI combined assay. Histograms represent the percentage of the treated cells subjected to the different death pathways, in comparison with control samples: early apoptosis (A), late apoptosis (B) and necrosis (C). *$p<0.05$, **$p<0.01$ and ***$p<0.001$.

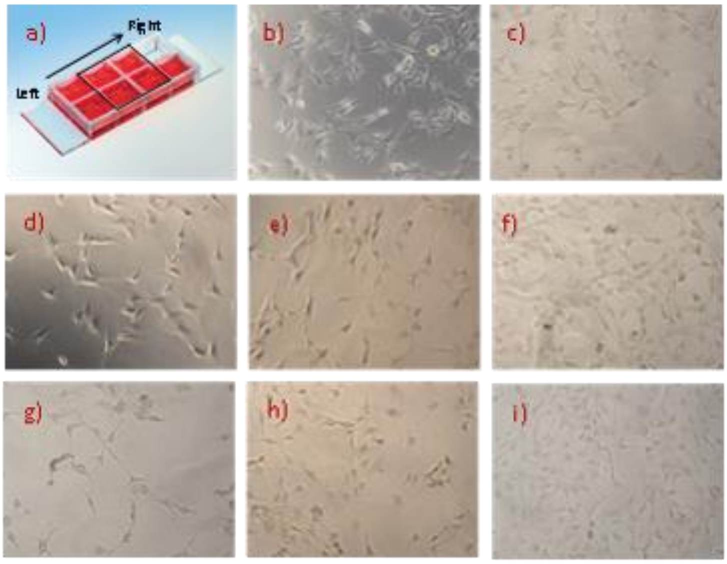

Figure S14: a) Images of the microscope slides used for the cleaved caspase-3 immunofluorescence experiments: only the central wells are affected by ultrasonic beam. The ulterior images represent photo captured in bright field just after treatments (from left to right in chamber slide): b) CTRL 15 min; c) CTRL 30 min; d) US 15 min; e) US 30 min; f) US NPs 15 min; g) US NPs 30 min; h) NPs 15 min; i) NPs 30 min.

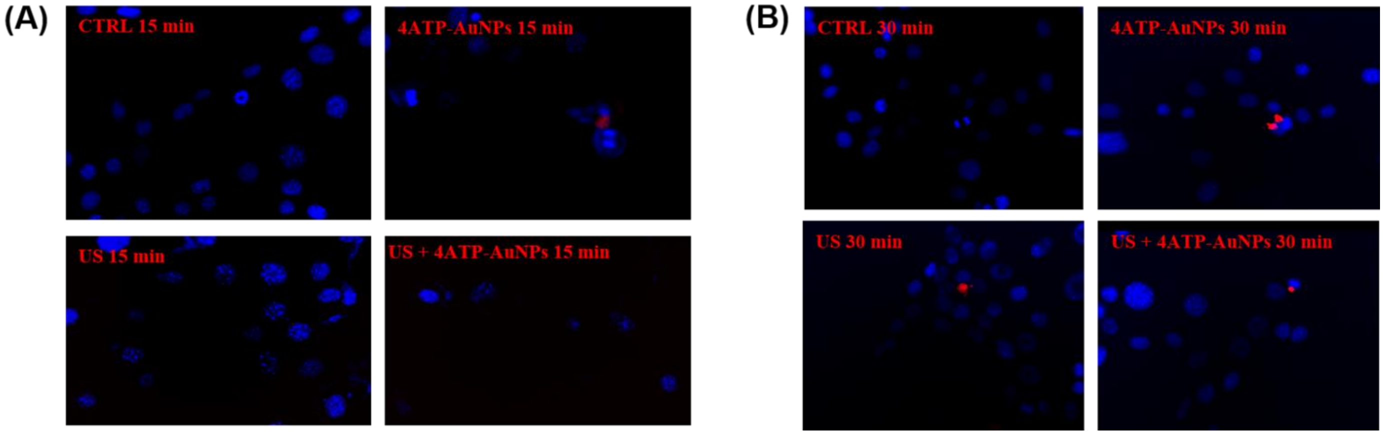

Figure S15: Immunofluorescence images of cleaved caspase-3 (red) and nuclei (blue) acquired on treated samples for 15 (A) and 30 (B) minutes. For each sample controls experiments have been performed on non-treated cells and on cells treated individually with the 4ATP-AuNPs nanoprobe and US.
